# Synthesis and Bioactivity Studies of Benzimidazole–Chalcone Hybrids

**DOI:** 10.3390/molecules31091546

**Published:** 2026-05-06

**Authors:** Herman D. Makgoathana, Siyanda T. Mthembu, Thandi V. Mhlanga, Mamoalosi A. Selepe, Molahlehi S. Sonopo

**Affiliations:** 1 Department of Chemistry, Faculty of Natural and Agricultural Sciences, University of Pretoria, Lynnwood Road, Hatfield, Pretoria 0002, South Africa; makgoathana.herman@gmail.com; 2 Department of Physical and Earth Sciences, Faculty of Natural and Applied Sciences, Sol Plaatje University, P/Bag x5008, Kimberley 8300, South Africa; siyanda.mthembu@spu.ac.za; 3 Department of Physiology, Faculty of Health Sciences, University of Pretoria, Gezina, Pretoria 0031, South Africa; thandi.mqoco@up.ac.za; 4 Applied Radiation Department, South African Nuclear Energy Corporation, SOC Ltd., Pelindaba, Brits 0240, South Africa

**Keywords:** α,β–unsaturated, pharmacological properties, benzimidazole–chalcone, bioactive compounds

## Abstract

Chalcones featuring α,β–unsaturated carbonyl group are associated with an extensive range of pharmacological properties. The synthesized derivatives of benzimidazole–chalcone are prominent classes of bioactive compounds that have demonstrated significant applications. Recently, there has been an encouraged demand for synthesizing aromatic *N*–heterocyclic α,β–unsaturated hybrids that comprise benzimidazole–chalcones, which could be evaluated for activities against several diseases. The current review presents the synthetic approaches of the recently prepared benzimidazole–chalcone compounds and their biological applications. The biological activity studies include cytotoxicity against several cancer cells, antibacterial, antifungal, antileishmanial, antimalarial, antiviral and antidiabetic activities. The in silico studies of hybridized benzimidazole–chalcones are also discussed. Therefore, the review shows the significance of benzimidazole–chalcone derivatives and their potential as effective bioactive agents.

## 1. Introduction

**Figure 1:**
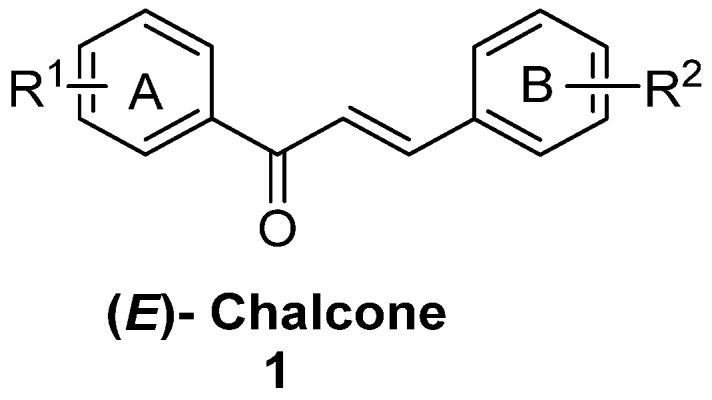
General structure of chalcones.

Chalcones are biogenetic precursors of flavonoids that consist of two aryl moieties bridged via an α,β–unsaturated carbonyl system (Figure 1) [1]. They are phenolic compounds, whose colours vary from yellow to orange and are naturally present in fruits, spices, teas, and soy-based meals [2]. Chalcones are regarded as privileged in the medicinal chemistry due to their potential beneficial properties [2,3]. They have remarkable biological activities such as antiproliferative [4], antifungal [5], antioxidant [6], antibacterial [7], antiviral [8], antileishmanial [9], antimicrobial [10], anti-inflammatory [11], anticancer [12], antimalarial [13], and many more. Synthetic chalcone analogues have been extensively studied based on their bioactive properties. Chalcones can serve as the intermediates, which can undergo chemical transformations involving conversion into heterocyclic compounds and other conjugated systems [14]. Furthermore, chalcones have been conjoined with other scaffolds to provide hybrids with improved biological activities [15]. Examples include fusing them with benzimidazoles [16].

**Figure 2:**
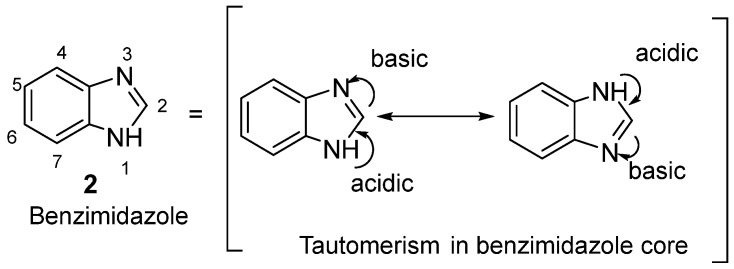
Benzimidazole resonance and its tautomeric formation.

Benzimidazole compounds are amongst the most widely used *N*-heterocyclic pharmacophores due to their wide occurrence in bioactive compounds and are used in drug development [17]. The first compound of the benzimidazole class was prepared by Hoebrecker in 1872, which was obtained as 2,5– or 2,6–dimethylbenzimidazole through reduction of 2–nitro–4–methylacetanilide derivative [18] and it was later re–synthesized by Ladenberg in 1875 [19]. The benzimidazole moiety **2** occurs as a result of benzene and imidazole ring system fusion at the 4 and 5 positions of the imidazole ring, with relative properties of amphoteric nature presenting with both acidic and basic properties due to the NH group [20]. Benzimidazoles occur in two equivalent tautomeric states (Figure 2) [21]. The benzimidazole nucleus is referred to as a “master key” compound since it serves as a significant core in multiple compounds that operate on various targets to produce a variety of therapeutic properties [22]. It shares some structural similarities with purine bases, and represents one of the classes of substances with the largest biological activities [23], including antibacterial [24], antimalarial [25,26], and anticancer [27].

**Figure 3:**
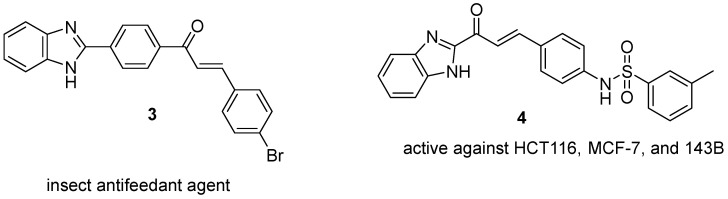
Hybridization of benzimidazolyl–chalcone derivatives.

Molecular hybridization of benzimidazoles and chalcones or other α,β–unsaturated carbonyl systems present attractive approach for the construction of bioactive derivatives. Several studies on fused benzimidazolyl–chalcone derivatives have yielded compounds with various biological properties. Examples include (*Z*)–1–(4–(1*H*–benzo[*d*]imidazol-2-yl)phenyl)–3–(4–bromophenyl)prop-2–en–1–one **3**, which act as insect antifeedant agent [28,29], and benzimidazole–chalcone hybrid **4** bearing sulfonamide moiety, which has been reported to exhibit effective activity against highly aggressive cell line (HCT116), Michigan Cancer Foundation–7 (MCF–7), and human osteosarcoma cell (143B) cancer cell lines (Figure 3) [30]. The present study highlights the synthetic approaches for benzimidazole–chalcone derivatives and demonstrates their biological activities, together with their significant contribution on various diseases. The databases that were used for the selection of the literature include Scifinder, Reaxys, Google Scholar and Scopus.

## 2. Benzimidazole Derivatives with Anticancer Activity

### 2.1. C-2-Substituted Derivatives with Anticancer Activity

**Figure 4:**
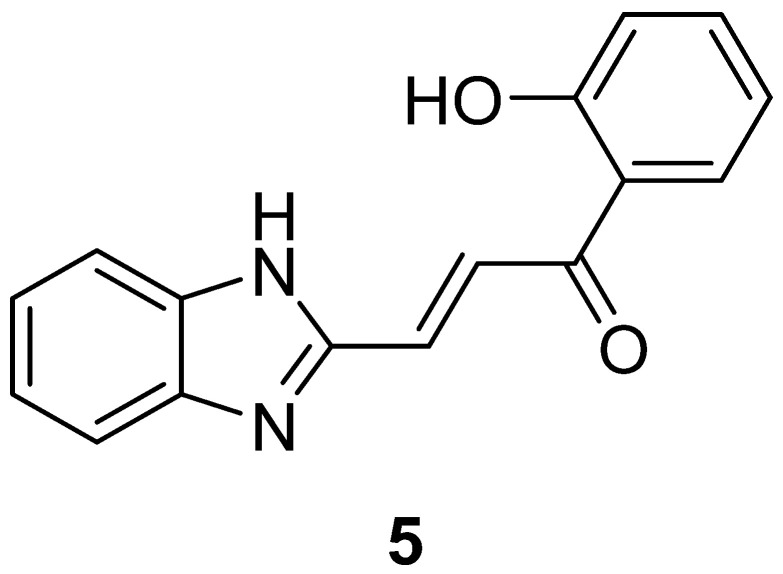
Benzimidazolyl–retrochalcone compound.

The substituted benzimidazole–chalcone derivatives have gained a substantial attention in the field of synthetic medicinal chemistry research as a promising anticancer agent, particularly the C–2–substituted benzimidazoles are playing an essential role by improving the biological activity of targeted compounds. In 2020, Mahama and colleagues reported the synthesis of 3-diphenyl–2–propen–1–one moieties by using a common synthetic approach, which involved the use of commercially available diamine compound as the starting material to synthesize benzimidazolyl–retrochalcones [31]. The synthesized benzimidazolyl–retrochalcone compounds were evaluated for their in vitro cytotoxic activity against a panel of cell lines such as immortalized human liver cancer (Huh–7), human prostate cancer (PC3), human epithelial lung cancer (NCI–H727), human colorectal adenocarcinoma (CaCo2), human colorectal carcinoma (HCT–116), human breast cancer (MDA–MB 231, MCF–7) and a normal fibroblast cell line and they showed cytotoxic activity with IC_50_ values ranging from 0.83 to 14.70 µM. Compound **5** was found to be more effective against colon cancer cells (HCT–116) having the IC_50_ value of 0.83 µM (Figure 4) [31].

**Scheme 1:**
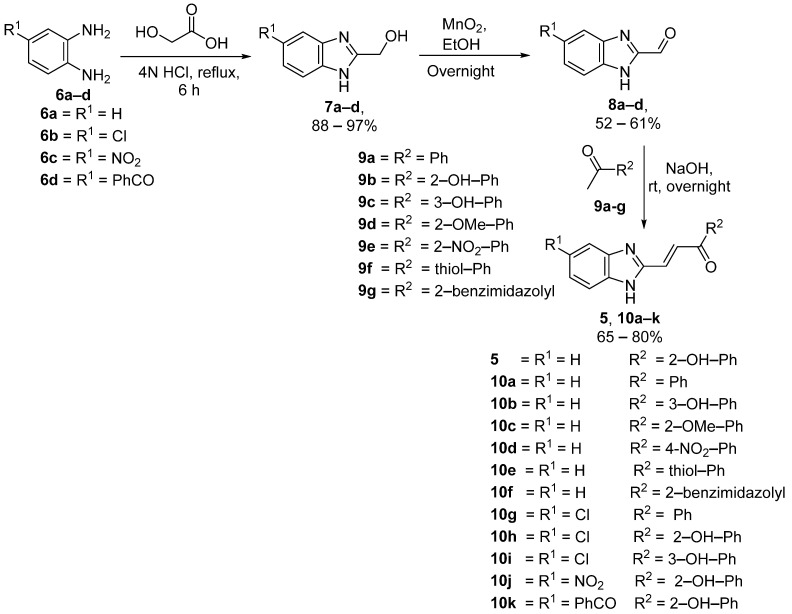
Synthesis of benzimidazole-based–retrochalcone derivatives.

In order to develop compounds with improved potency that can overcome mechanisms of resistance, Koné et al. took advantage of molecular hybridization of chalcones and benzimidazoles. They reported the synthesis of the benzimidazole-based retrochalcone derivatives according to Scheme 1 [32]. The commercially available *o*-phenylenediamines **6a**–**d** were reacted with glycolic acid under acid reflux following the Phillips method to offer (1*H*–benzimidazol–2–yl)methanols **7a**–**d** [32,33]. Compounds **7a**–**d** were subjected to oxidation, using activated manganese oxide to produce 1*H*-benzimidazole-2-carbaldehydes **8a**–**d**. The requisite 3-benzimidazolyl–retrochalcones **5** and **10a**–**k** were obtained from the reaction of **8a**–**d** with the appropriate acetophenones **9a**–**g**. The synthesized compounds were initially assessed for the cytotoxicity, and they showed activity against fibroblasts with IC_50_ values ranging from 0.61 to 9.29 μM and against human lung cancer cells (NCI–H727) with the IC_50_ values ranging from 1.20 to 8.63 μM [34]. Recently (2025), compounds **5** and **10a**–**f** were evaluated for their biological properties [33], and they demonstrated significant anticancer activity against both breast (MDA–MB–231 and MCF–7), and colon (Caco–2 and HCT–116) cancer cell lines. The IC_50_ values ranged between 0.06 μM and 12.60 μM. Compound **10c** emerged as the most potent, having IC_50_ values lower than 1.56 μM in all cell lines tested and compound **10d** was found to be inactive against both Caco–2 and MDA–MB–231 cell lines. Compounds with electron–donating groups were found to be highly potent, while those with electron–withdrawing groups showed poor potency [33].

**Scheme 2:**
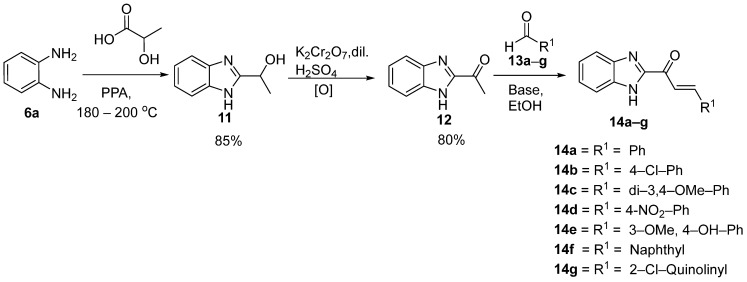
Synthesis of the benzimidazole–chalcone series.

Uthale et al. (2023) synthesized benzimidazole–chalcone conjugates by condensation of **6a** with 2-hydroxypropanoic acid in polyphosphoric acid (PPA) to afford compound **11** that was subsequently oxidized by potassium dichromate to achieve compound **12** (Scheme 2) [35]. Compound **12** was reacted with various aldehydes **13a**–**g** to offer compounds **14a**–**g** [35].

The synthesized compounds **14a**–**g**, which were designed as dual inhibitors for Bcl–2 and Mcl–1 showed significant binding interactions of the anti–apoptotic proteins. They were also screened for their in vitro cytotoxic activity against two oral cancer cell lines, AW8507 and AW13516. Compound **14g** exhibited significant cytotoxic activity with the IC_50_ values of 7.89 and 7.12 μM against AW8507 and AW13516 cell lines as indicated in Table 1. In this series, the electron–withdrawing nitro group on the phenyl ring was beneficial for the activity, while the electron-donating methoxy and hydroxy substituents were detrimental for the activity [35].

**Scheme 3:**
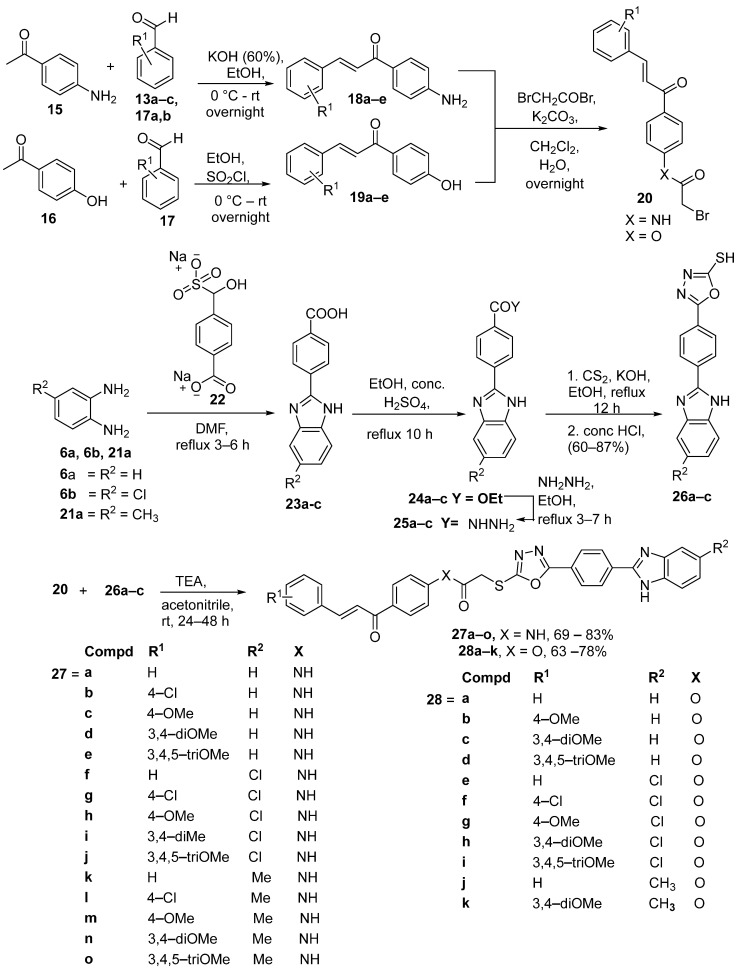
Synthesis of 1,3,4-oxadiazole–chalcone/benzimidazole derivatives.

Hagar et al. (2023) accomplished the synthesis of the 1,3,4–oxadiazole–chalcone–benzimidazole hybrids aiming for synergistic antiproliferative activities from the three scaffolds (Scheme 3) [36]. The synthesis was initiated by reacting to the 1–(4–aminophenyl)ethanone (**15**) with the substituted benzaldehyde derivatives **13a**–**c**, **17a**,**b** to offer compounds **18a**–**e**, and 1–(4–hydroxyphenyl)ethanone (**16**) was reacted with benzaldehydes **13a**–**c**, **17a**,**b** to offer compounds **19a**–**e**. Alkylation of compounds **18** and **19** afforded compounds **20**. On the other hand, the oxadiazole precursors **26a**–**c** were synthesized via step-by-step reactions starting from diamines **6a**, **6b** and **21a**. Condensation of **6a**, **6b**, and **21a** and sodium (4–carboxyphenyl)(hydroxy)methanesulfonate (**22**) under reflux gave 4–(1*H*–benzo[*d*]imidazol–2–yl)benzoic acid derivatives **23a**–**c**. Esterification of **23a**–**c** rendered **24a**–**c**, which was reacted with hydrazine hydrate followed by carbon disulphide to afford compounds **26**. The targeted benzimidazole–chalcones **27a**–**o** and **28a**–**k** were prepared by the condensation of **20** and **26a**–**c** in the presence of triethylamine [36].

The synthesized compounds **27a**–**o** and **28a**–**k** were screened for the antiproliferative activities, and their results are shown in Table 2 and Table 3. Derivatives with chloro substituent on the benzimidazole core (**27f**–**27j**) displayed potent antiproliferative activity, where **27h** showed better activity [IC_50_ (µM) A–549 (1.30 ± 0.50), MCF–7 (0.80 ± 0.08), Panc–1 (1.20 ± 0.20), and HT–29 (1.13 ± 0.20)]. The active compounds were further evaluated for inhibition of epidermal growth factor receptor (EGFR) and B–raf proto-oncogene (BRAF). Compounds **27g**, **27h**, and **27i** exhibited activity against EGFR with IC_50_ values of 2.2 ± 1.2, 1.8 ± 0.7, and 3.6 ± 0.8 µM, respectively; and 8.9 ± 3.7, 4.6 ± 1.7, and 5.8 ± 1.5 µM against BRAF, respectively. Furthermore, the active hybrids were subjected to molecular modelling studies to evaluate their binding mode in the ATP binding site of EGFR [36].

**Scheme 4:**
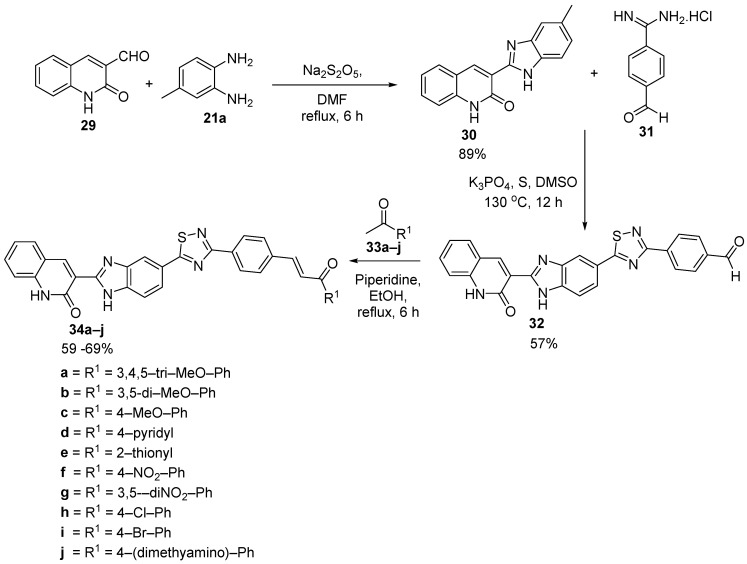
Synthesis of 1,2,4–thiadiazol–benzo[*d*]imidazol–2–yl)quinolin–2(1*H*)–one.

The preparation of 2–quinoline–benzimidazole–1,2,4–thiadiazoles was achieved as shown in Scheme 4 [37]. The synthesis was initiated by reacting 1,2-dihydro–2–oxoquinoline–3–carbaldehyde (**29**) with 4–methylbenzene–1,2–diamine (**21a**) through oxidative-cyclization under reflux to give compound **30**. The produced compound **30** was treated with 4-formylbenzamidine hydrochloride (**31**) and elemental sulphur (S) in DMSO at elevated temperatures (130 °C) in basic conditions (K_3_PO_4_) to give benzo[*d*]imidazol–5–yl)–benzaldehyde (**32**). Compound **32** was subjected to an aldol condensation reaction with various acetophenones **33a**–**j** to offer the 2-quinoline–benzimidazole–1,2,4–thiadiazoles compounds **34a**–**j**.

The compounds **34a**–**j** were evaluated for anticancer activity against four different human cancer cell lines, MCF–7, A549, Colo-205 and A2780 using the clinical drug candidate etoposide as a reference. The compounds showed activity with IC_50_ values ranging from 0.012 ± 0.007 to 13.3 ± 5.46 µM. Compound **34d** with pyridyl group was the most active against MCF–7, A549, Colo–205 and A2780 cell lines with IC_50_ value of 0.012 ± 0.007 µM, 0.074 ± 0.004 µM, 1.46 ± 0.73 µM, and 0.083 ± 0.002 µM, respectively [37]. Structure Activity relationship (SAR) study showed that the compound with 3,4,5–trimethoxyphenyl scaffold on the chalcone **34a** (MCF–7 = 0.16 ± 0.054 µM; A549 = 0.26 ± 0.053 µM; Colo-205 = 0.37 ± 0.082 µM and A2780 = 1.46 ± 0.54 µM) was more potent than the compound with dimethoxy substituents **34b** (MCF–7 = 1.99 ± 0.75 µM; A549 = 0.79 ± 0.022 µM; Colo-205 = 1.58 ± 0.68 µM and A2780 = 1.28 ± 0.65 µM), while monomethoxy-substituted compound **34c** showed lesser activity on all cell lines (MCF–7 = 2.34 ± 1.74 µM; Colo–205 = 7.48 ± 3.99 µM and A2780 = 6.44 ± 2.64 µM). This implied that the activity decreased with the decreasing number of methoxy substituents on the chalcone nucleus. Substitution of the 4–methoxy group on the chalcones with electron–withdrawing groups such as 4–nitro, 4–chloro, and 4–bromo groups led to moderate activities. Compound **34g** with two nitro groups on the chalcone showed slightly increased activity (MCF–7 = 0.01 **±** 0.005 µM; A549 = 0.88 ± 0.068 µM; Colo-205 = 0.22 ± 0.018 µM and A2780 = 0.19 ± 0.085 µM) in comparison to **34f**, **34h** and **34i**. Compound **34j** with the weaker electron-donating dimethylamino group displayed the lowest potency [37].

### 2.2. C–2 and N–Substituted Derivatives

**Scheme 5:**
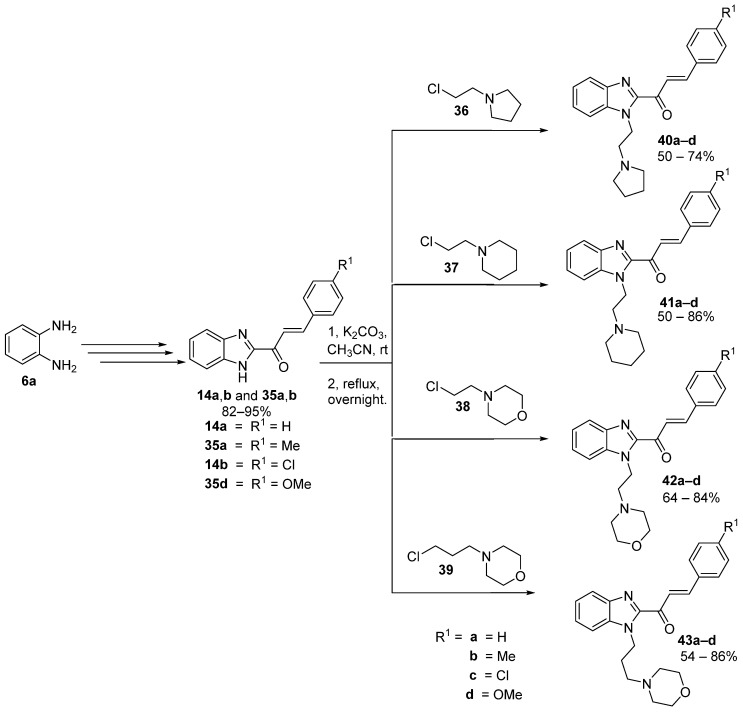
Alkylated benzimidazole–chalcone derivatives.

The C–2 and *N*–benzimidazole–chalcone scaffolds have shown potential antimicrobial, anticancer, anti-inflammatory, and enzyme-inhibitory activities [38,39]. Hsieh et al. (2019) designed and synthesized the benzimidazole–chalconederivatives, as part of an open-chained flavonoid family featuring an α,β–unsaturated carbonyl (Scheme 5) [40]. The 1–(1*H*–benzo[*d*]imidazol–2–yl)ethanol **11** was obtained by refluxing *o*–phenylenediamine (**6a**) with 2–hydroxypropanoic acid in hydrochloric acid. Compound **11** was oxidized with potassium permanganate and solid aluminum oxide to provide the intermediate compound **12**. The benzimidazolyl-chalcones **14a**,**b** and **35a**,**b** were obtained from the reaction of **12** with the substituted aromatic aldehydes **13a**,**b** and **34a**,**b** under basic conditions. The alkyl–substituted benzimidazolyl-chalcones **40a**–**d** to **43a**–**d** were obtained from the reaction of various alkyl chlorides **36**–**38** with **14a**,**b**, **and 35a**,**b**. The synthesized compounds **40**–**43** were screened against human ovarian cancer cell line (OVCR-3). The compounds **40**–**43** exhibited antiproliferative activity against OVCR–3 cell line with the IC_50_ values of 10.50 (**40a**), 10.34 (**41a**), 22.44 (**42a**), and 10.76 μM (**43a**). Compound **43a** was the most active compound among the tested benzimidazole derivatives, which exhibited IC_50_ values of 9.73, 8.91, 10.93 and 10.76 μM on the A549, MCF–7, HEP–G2 and OVCAR–3 cells, respectively. This compound showed in vitro cytotoxicity comparable or superior to that of cisplatin. Furthermore, other synthesized compounds were assessed for the antiproliferative effects on various cell lines, and showed growth inhibitory capability on an immortalized human liver cancer cell line (HEP–G2), human alveolar basal epithelial cell line (A549), OVCAR–3, and immortalized human breast cancer (MCF–7) cell lines with IC_50_ values ranging from 10.34 to 14.88 μM [40]. The SAR study showed that compounds bearing nitrogen containing 5– or 6–memebered rings with hydrocarbon spacer groups (**40a**, **41a** and **43a**) were more active than the non–substituted benzimidazole–chalcone **14a** on A549, MCF–7, and OVCAR–3 cells. In addition, the compounds **40a**, **41a** and **43a** showed greater potency on all cell lines tested than the corresponding compounds substituted with the 4–methyl–, 4–methoxy– and 4–chloro– substituents on phenyl ring (**40b**–**40d**, **41b**–**41d**, and **43b**–**43d**) [40].

**Scheme 6:**
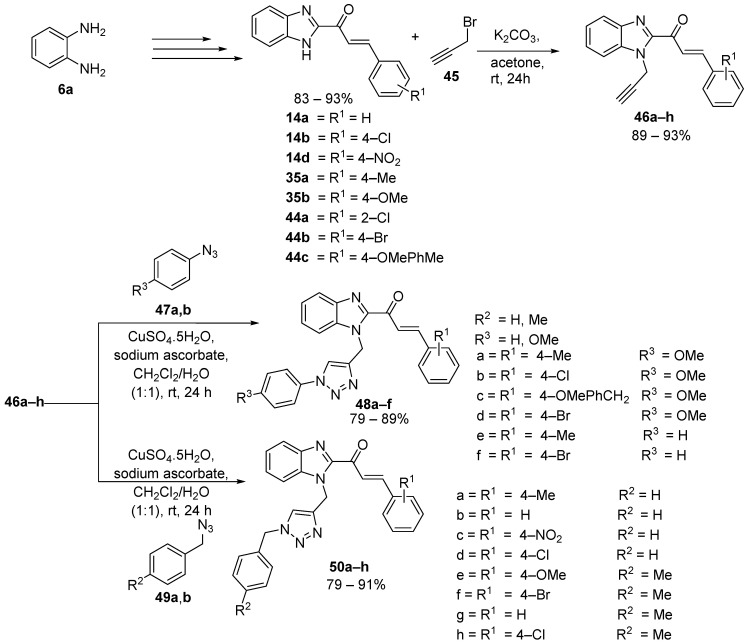
Substituted triazole–benzimidazole–chalcone derivatives.

Djemoui et al. (2020) reported the successful synthesis of *N*–substituted triazole–benzimidazole–chalcones **48a**–**f** and **50a**–**h**, which were achieved via a sequential approach that was initiated by condensation of **6a** until the synthesis of the benzimidazole–chalcone derivatives **14a**,**b**,**14d**, **35a**,**b**, **44a**–**c** using common method as highlighted previously (Scheme 6) [41]. Compounds **14a**,**b**,**14d**, **35a**,**b**, **44a**–**c** were further reacted with propargyl bromide **45**, producing *N*–propargylated compounds **46a**–**h**, and the next step involved subjecting **46a**–**h** to click chemistry conditions thereby offering 1,2,3–triazole–benzimidazole–chalcones by using azide compounds **47** and **49** to afford the targeted compounds **48a**–**f** and **50a**–**h**, respectively. The synthesized compounds were screened for antiproliferative activity and the chloro–substituted compounds **50d** and **50h** demonstrated enhanced cytotoxic effects. Compound **50d** showed activity against breast cancer cell line infiltrating ductal carcinoma (T47-D) and MDA–MB–231 (IC_50_ = 6.23 ± 1.03 µM and 5.89 ± 1.35 µM, respectively) and **50h** exhibited activity against prostate cancer cell line PC3 (IC_50_ = 5.64 ± 1.33 µM). The results of **50d** and **50h** signify that the chloro substituent on the chalcone moiety was essential for the activity [41].

**Scheme 7:**
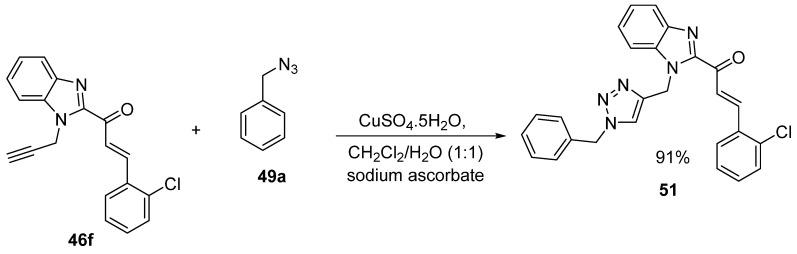
Synthesis of triazole–benzimidazole–chalcone hybrid.

Farag et al. (2025) also synthesized triazole–benzimidazole–chalcone hybrid (*Z*)–1–(1–((1–benzyl–1*H*–1,2,3–triazol–4–yl)methyl)–1*H*-benzo[*d*]imidazol–2–yl)–3–(2–chlorophenyl)prop–2–en–1–one (**51**) from the benzimidazole alkyne **46f** and pre prepared (azidomethyl)benzene **49a** (Scheme 7) [38]. Compound **51** was screened for antiproliferative activity. It exhibited activity with IC_50_ values of 6.23 ± 1.03, 5.89 ± 1.03, and 10.7 ± 1.03 µM against T47-D, MDA–MB-231, and PC3 cancer cells, respectively [38].

**Scheme 8:**
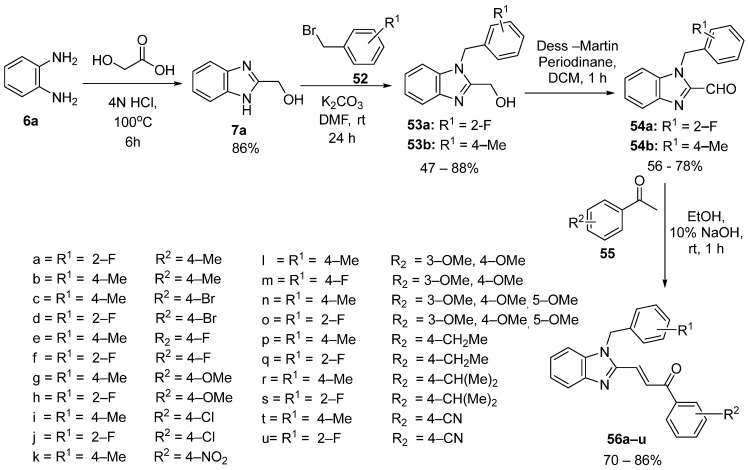
Benzimidazole–chalcone derivatives as Topo II inhibitors.

Zhou et al. synthesized benzimidazole–chalconehybrids (Scheme 8). Compound **7a** was obtained through refluxing of **6a** with glycolic acid in hydrochloric acid. *N*–Alkylation of **7a** with benzyl bromides **53** under basic conditions gave substituted compounds **53a** and **53b**. Compounds **53a** and **53b** were subjected to oxidation using Dess–Martin reagent to yield the substituted (1-benzyl–1*H*-benzo[*d*]imidazol–2–yl)–aldehydes **54a** and **54b**. The final compounds **56a**–**u** were prepared by the reaction of **54a** and **54b** with the acetophenones **55** under the Claisen–Schmidt reaction conditions [42,43].

The synthesized compounds **56a**–**u** were determined to effectively inhibit Topoisomerase II. Compounds with an electron-donating groups such as (–C(CH_3_)_2_, –CH_2_CH_3_,–CH_3_, –OCH_3_) at R^2^ position showed pronounced inhibitory activity on Topo II than the compounds with an electron–withdrawing groups (–NO_2_, –CN). Compound **56n** was identified as the most active compound, which inhibited 94.9% at 20 µM and 93.1% at 10 µM of the Topo II’s catalytic activity in DNA relaxation, compared with the positive control group without Topo II. The compounds were also evaluated for antiproliferative effects on four cancer cells, including HepG2 (human hepatoma cell line), A549 (human lung cancer cell line), LNCaP (human prostate cancer cell line), MG63 (human osteosarcoma cell line). The most active compounds were **56a** and **56h**, which exhibited activity of 2.8 ± 0.2 and 2.9 ± 0.2 µM, respectively A549 cancer cell. The most active Topo II inhibitor 57n exhibited activity of 3.8 ± 1.8, 4.6 ± 0.2, 4.1 ± 0.6, 3.6 ± 0.3 µM on A549, HepG2, MG63 and LNCaP cell lines, respectively [42,43].

**Figure 5:**
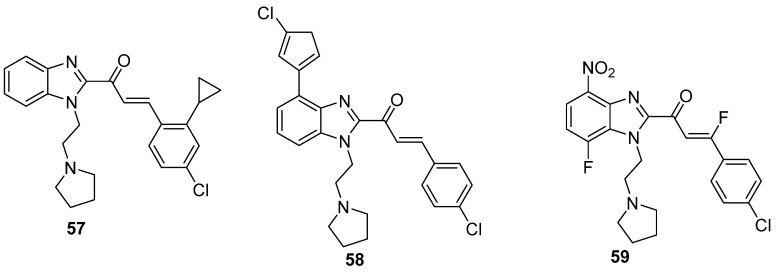
Substituted-benzimidazole–chalconeligands.

With the aim of applying computer-aided techniques to design highly potent and less toxic benzimidazole–chalcone-based chemotherapeutic agents against hepatocellular carcinoma by targeting the EGFR receptor, Ameji et al. (2023) conducted the outcomes of the theoretical simulations for the benzimidazole–chalconeagainst HepG2 cancer cell line [44]. Structural optimization led to the identification of ligands **57**, **58**, and **59** (Figure 5) as active compounds, with predicted IC_50_ values of 26.07, 43.38, and 178.69 μM, respectively [44]. The compounds were further subjected to molecular docking against the active sites of EGFR. protein target. The binding energy values of −8.4, −8.9, and −8.1 were obtained for **58**, **59** and **60**, respectively [44].

**Scheme 9:**
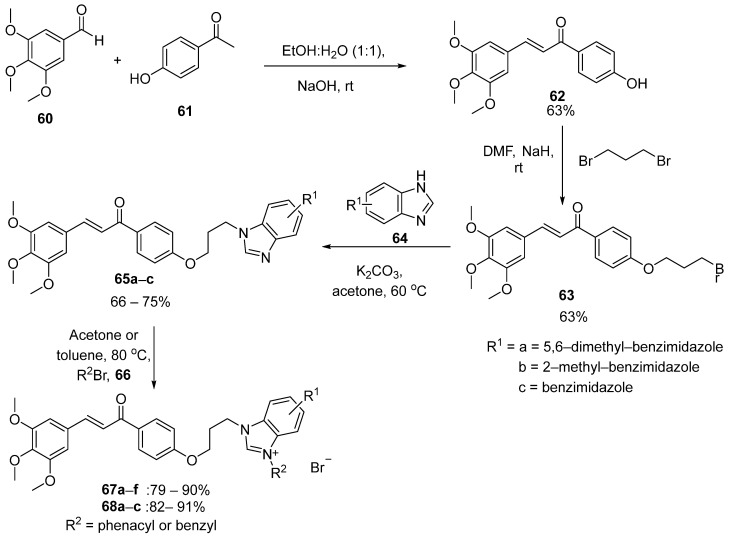
Syntheses of (*E*)–1–(4–(3–(1*H*–benzo[d]imidazol–1–yl)propoxy)phenyl)–3–(3,4,5–trimethoxyphenyl)prop–2–en–1–one hybrid compounds.

Yang et al., (2019), reported the synthesis of trimethoxyphenyl–derived chalcone–benzimidazolium salts [45]. The synthesis was started by reacting 3,4,5–trimethoxybenzaldehyde (**60**) with 1–(4–hydroxyphenyl)ethan–1–one (**61**) under basic conditions offering (*E*)–1–(4–hydroxyphenyl)-3-(3,4,5-trimethoxyphenyl)prop–2-en–1–one (**62**). The (*E*)–chalcone derivative **62** was alkylated with 1,3–dibromopropane in the presence of NaH to produce compound (*E*)–1–(4–(3–bromopropoxy)phenyl)–3–(3,4,5–trimethoxyphenyl)prop–2–en–1–one (**63**). The compound **63** was reacted with 1*H*–benzo[*d*]imidazoles **64** in the presence of K_2_CO_3_ using acetone as a solvent to give the benzimidazole–chalcones **65a**–**c**. The compounds **65a**–**c** were further treated with phenacyl bromide or benzyl bromide (**66**) yielding benzimidazole–chalconesalts **67a**–**f** and **68a**–**c** (Scheme 9) [45].

The synthesized compounds **65a**–**c**, **67a**–**f**, and **68a**–**c** were evaluated for the in vitro activity against a panel of human tumour cell lines such as leukemia (HL–60), myeloid liver carcinoma (SMMC–7721), lung carcinoma (A549), breast carcinoma (MCF–7), and colon carcinoma (SW480) where Cisplatin (DDP) was used as the reference drug (Table 4). The benzimidazole–chalcone**65a**–**c** showed good activity for HL-60, SMMC–7721, A–549, MCF–7, and SW480 cell lines. The compounds **67d** and **67f** were found to be more selective to HL-60 cell lines with IC_50_ values of 0.83 and 0.59 μM, respectively. The results show that the 5,6–dimethyl–benzimidazole or 2–methyl–benzimidazole ring as well as the 2–naphthylmethyl, 4–methylbenzyl, or 2–naphthylacyl substituent at position–3 of the benzimidazole ring was important to the cytotoxic activity [45,46].

**Scheme 10:**
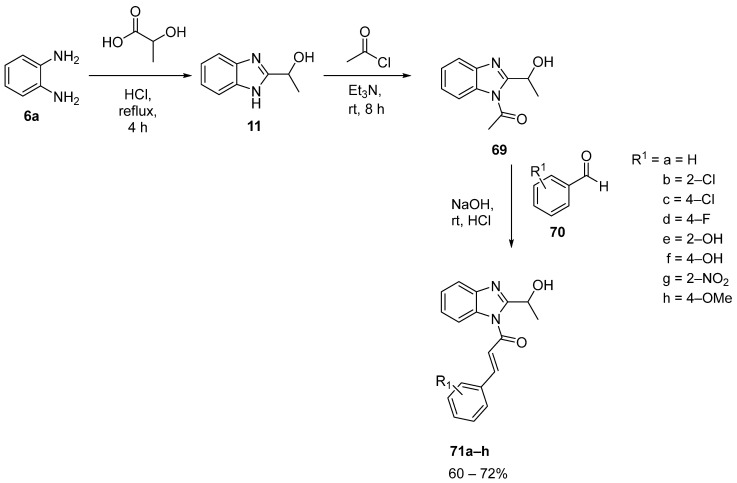
Synthesis of 2–(1–hydroxy–ethyl)–1*H*–benzimidazol–1–yl–chalcone derivatives.

Chhajed et al. described a library on the synthesis of 3–(substitutedphenyl)–1–[2-(1–hydroxy–ethyl)]–1*H*–benzimidazol–1-yl)–chalcone derivatives as depicted in Scheme 10. The 1–(1*H*–benzo[*d*]imidazol–2–yl)ethanol (**11**) were reacted with acetyl chloride to offer 1–(2–(1–hydroxyethyl)–1*H*–benzo[*d*]imidazol–1–yl)ethanone (**69**). The benzimidazole–chalcones **71** (Scheme 10) were obtained from the treatment of **69** with substituted benzaldehydes **70**. The benzimidazole–chalcone**71a**–**h** were evaluated for their EGFR antagonism through the molecular docking analysis. The synthesized compounds were also evaluated for their in vitro anticancer activity by propidium iodide fluorescent assay and Trypan blue viability assay against colorectal cancer cell lines (HCT116) and non-small lung cancer cell lines (H460). The cytotoxic studies have shown that the compound **71a** gave the IC_50_ = 7.31 and 10.16 µM against HCT116 and H460 cell lines, respectively, by PI assay and compound **71h** showed the IC_50_ = 12.52 and 6.83 µM against HCT116 and H460 cell lines, respectively [46].

## 3. Benzimidazole–Chalcones with Antimicrobial Activity

### 3.1. C-2-Substituted Derivatives with Antimicrobial Activity

**Scheme 11:**
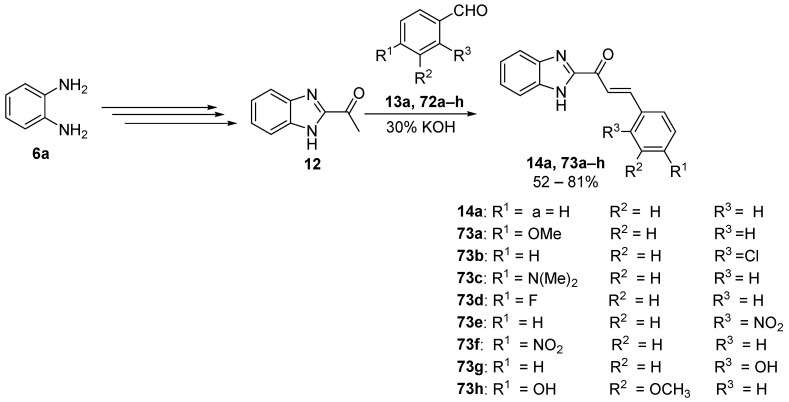
Synthesis of chalcone-derived benzimidazoles **14a**, **73a**–**h**.

The C-2-substituted benzimidazoles have proven to be compounds with improved antimicrobial potency. Babu and Selvakumar have synthesized benzimidazole–chalcones, which were obtained from the reaction of *o*–phenyldiamine (**6a**) with 2–hydroxypropanoic acid to give 1–(1*H*–benzo[*d*]imidazol–2–yl)ethanol (**11**), which was further oxidized with 20% K_2_Cr_2_O_7_ to offer 1–(1*H*–benzo[*d*]imidazol–2–yl)ethanone (**12**). Compound **12** was reacted with various substituted benzaldehydes **13a**, **72a**–**h** to produce the benzimidazole–chalcones **14a**, **73a**–**h** (Scheme 11). The final compounds were evaluated for antimicrobial activities using disc diffusion and micro broth dilution assays and compounds **73a**, **73d**, **73e** and **73f** showed effective zone of inhibition against *Klebsiella*, and exerted potent in vitro antifungal activity against *Aspergillus*, *Penicillium* and *Candida* species. On the other hand, compounds **14a**, **73a**, and **73h** had better activity against *Aspergillus*, *Penicillium* and *Candid*a species [47].

**Scheme 12:**
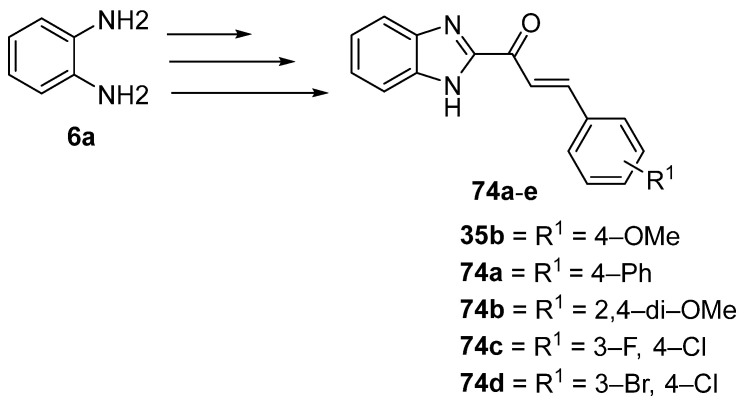
Synthesized hybridized benzimidazole–chalconecompounds.

Shahin et al. reported the synthesis of the hybridized benzimidazole–chalconederivatives, which were initially obtained from the *o*–phenyldiamine (**6a**) until 1–(1*H*–benzo[*d*]imidazol–2–yl)ethanone (**12**), which were further reacted with various aldehydes to yield **35b**, **74a**–**d** (Scheme 12) [48].

The compounds **74a**–**d** were screened for antimicrobial activity against *Escherichia coli*, *Staphylococcus aureus*, and three clinical isolates of linezolid-resistant MRSA. It was observed that the initial screening, which was performed on *S*. *aureus* and *E*. *coli* showed moderate inhibitory activity of microbial growth with MIC values around 5 mg/mL for compounds **74a** and **74b** towards *S. aureus*, and compound **74d** was found to be the most promising antimicrobial agent with MIC 3.05 mg/mL against standardized, methicillin-resistant *Staphylococcus aureus* bacteria (MRSA ATCC 43300). Furthermore, in silico ADME study, which was performed to confirm that the synthesized structures have acceptable Mwt, clogP, and PSA values, was achieved using Accelrys Discovery Studio 2.5 to forecast the ADME of the targeted compounds [48].

**Scheme 13:**
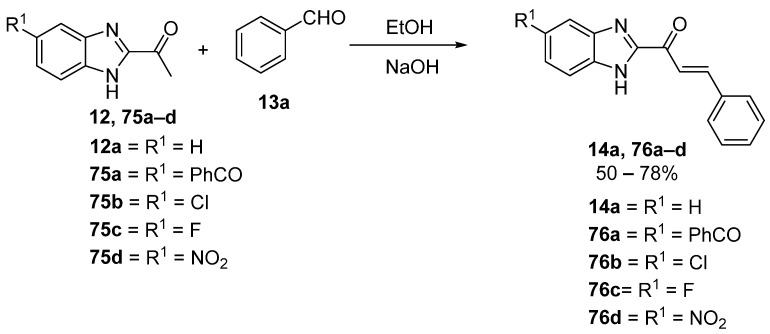
Synthesis of benzimidazolyl–chalcone hybrids from 2–acetylbenzimidazole.

Songuigama et al., reported the synthesis of benzimidazolyl-chalcone hybrids **14a**, **76a**–**d** achieved from the reaction of the substituted 2–acetylbenzimidazoles **75** treated with benzaldehyde (1**3a**) (Scheme 13) [49]. The synthesized compounds were evaluated for the antifungal activity, which showed that three derivatives exhibited anti candidiasis activity at the threshold quantity of 10 μg (Table 5). The activity was observed on the compounds **14a**, **76c**, and **76d**, which were active on the *Candida* strain with concentrations ranging from 5 to 1.25 μg/mL. The results showed that the halogen substituents (Chloro and fluoro) on the benzimidazole nucleus were vital for the activity. The non–substituted **14a** was also active [49].

**Scheme 14:**
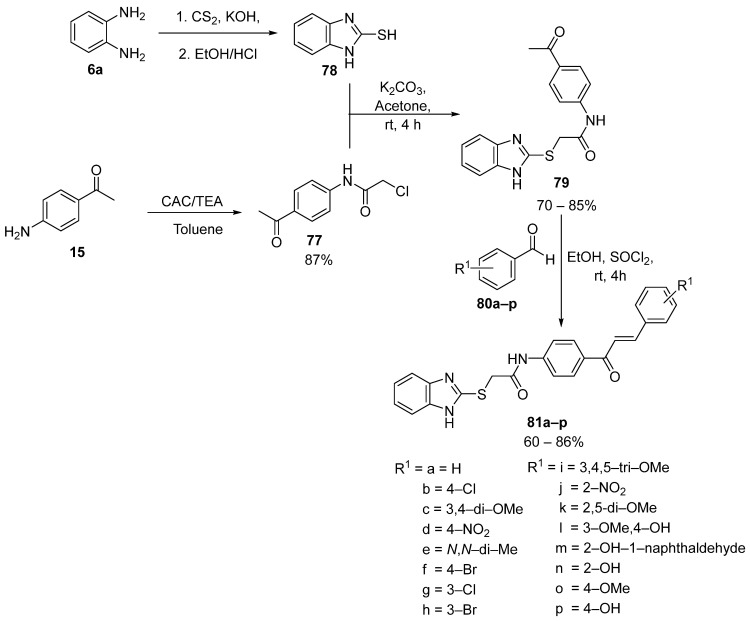
Synthesis of benzimidazole-clubbed chalcone derivatives.

Parikh et al. described the condensation reaction of acetophenone-bearing benzimidazole and various substituted aldehydes via the acid catalyzed process yielding the chalcone-possessing benzimidazole as a prime motif (Scheme 14). The initial process was started by reacting to the *o*–phenylenediamine (**6a**) with carbon disulfide under acid conditions, yielding 2–mercapto–benzimidazole **78**. The 1–(4–aminophenyl)ethanone (**15**) was reacted with chloroacetyl chloride to offer an intermediate *N*-(4-acetylphenyl) 2–chloroacetamide **77**, which was reacted with **78** to produce the 2–(1*H*–benzo[*d*]imidazol–2–ylthio)–*N*-(4–acetylphenyl)acetamide (**79**). Compound **79** was reacted with differently substituted aldehydes **80a**–**p** via acid–catalyzed aldol condensation reaction yielding substituted–2–(1*H*–benzo[*d*]imidazol-2-ylthio)–*N*–(4–((*E*)–3–phenylacryloyl)phenyl)acetamides **81a**–**o** (Scheme 14).

The synthesized compounds were evaluated for antimicrobial activities against Gram-positive bacteria, Gram–negative bacteria, and fungal strains. The compounds exhibited antifungal activity against *Candida albicans* and *Aspergillus niger* with MIC values ranging from 62.5 to 250 µg/mL. They also exhibited activity against Gram–positive bacteria *S*. *aureus* and *E*. *faecalis* with MIC values of 15.62- 250 µg/mL. Compounds with both electron–donating and electron–withdrawing group such as the derivatives with substitutions like *p*–NO_2_, *m*–Cl, 2,5-diOCH_3_, *o*–OH, and *p*–OH exhibited good activity against Gram–positive bacteria, particularly *S. aureus* compared with standard drug, and compound **83L** was the most active with MIC of 15.62 µg/mL against *S. aureus*. The same trend was observed for the Gram–negative bacteria, especially against *E. coli*. The compounds **81c**, **81i**–**k** and **81n** exhibited activity against *E. coli* with MIC value of 31.25 µg/mL [50].

### 3.2. N-Substituted Derivatives

**Scheme 15:**
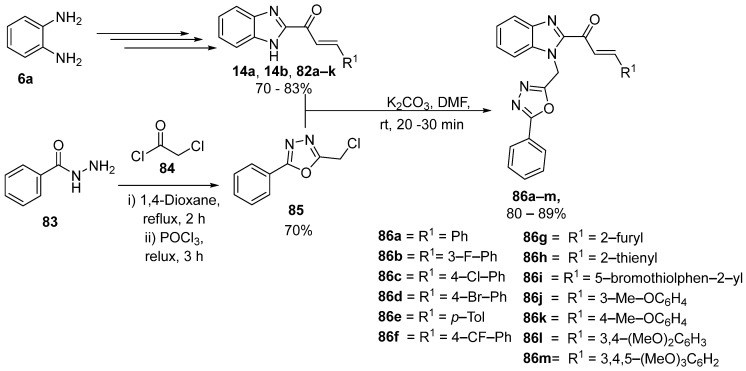
Synthesis of benzimidazole-derived chalcones containing 1,3,4-oxadiazole moiety.

The *N*–1 and –3 substituted benzimidazole–chalcone derivatives have shown broad biological activities including antimicrobial activities. Meshram and Vala have reported the synthesis of benzimidazole–chalcones containing the 1,3,4-oxadiazole moiety, which were obtained from the *o*–phenylene diamine (**6a**) and 2–hydroxypropanoic acid, yielding intermediate **11**. Compound **11** was subjected to oxidization with K_2_Cr_2_O_7_ to offer **12**. The benzimidazole–chalcones **14a**, **14b** and **82a**–**k** were prepared through the Claisen–Schimdt condensation reaction of the substituted aromatic aldehyde and **12** (Scheme 15). Compound **83** was reacted with 2–chloroacetyl chloride (**84**) to give 2–(chloromethyl)-5-phenyl–1,3,4–oxadiazole (**85**), which was reacted with benzimidazoles **14a**, **14b** and **82a**–**k** under basic conditions to yield the targeted 1,3,4–oxadiazole–benzimidazole–chalcones **88a**–**m** [51]. The synthesized compounds **14a**, **14b** and **82a**–**k** and **86a**–**m** were screened for antimicrobial activity against Gram–negative bacteria, Gram–positive bacteria and fungi. The compounds **86e**, **86g**, **86d**, and **88c** exhibited potent inhibitory activity (MIC = 150, 150, 100, and 125 µM, respectively) against *E*. *coli*, *P*. *aeruginosa*, *S*. *aureus*, and *S*. *pyogenes*, respectively, compared to standard drugs chloramphenicol (MIC = 150, against *E. coli*, *P. aeruginosa*, *S. aureus*, and *S. pyogenes*,) and ampicillin (MIC = 150, 750, 250 µM against *E*. *coli*, *S*. *aureus*, and *S*. *pyogenes*, respectively). For the antifungal activity, compounds **86b**, **86d**, **86g**, **86l**, **88m** gave a higher activity than the standard drug griseofulvin against *C albicans*. Overall, the synthesized compounds exhibited activities with MIC values ranging from 125 to 2000 µM [51].

**Scheme 16:**
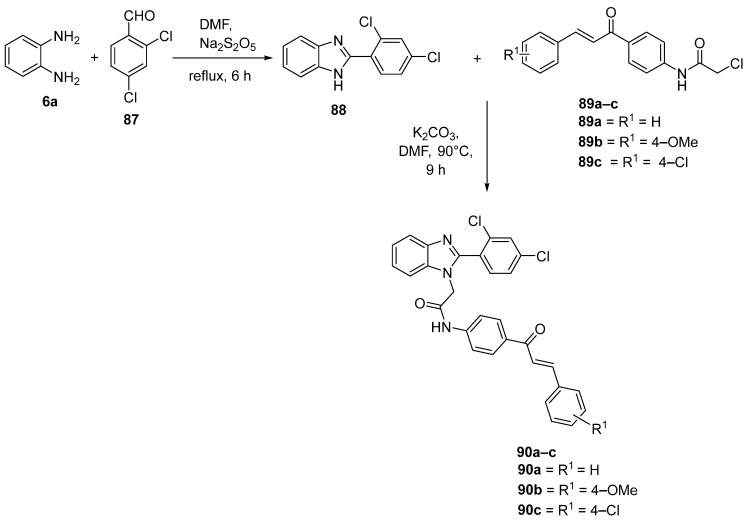
Synthesis of substituted 2–(2–(2,4–dichlorophenyl)–1*H*–benzo[d]imidazol–1-yl)–*N*–(4–((*E*)–3–phenylacryloyl)phenyl)acetamide.

Ibrahim et al., (2025), prepared a library of benzimidazoles bearing the chalcone moiety following the route shown in Scheme 16, where *o*–phenylenediamine (**6a)** was condensed with 2,4–dichlorobenzaldehyde (**87)** under reflux to offer 2–(2,4–dichlorophenyl)–1*H*–benzo[*d*]imidazole (**90**) [52]. Compound **88** was subjected to coupling with 2–chloro–*N*–(4–((*E*)–3-phenylacryloyl)phenyl)acetamide **89a**–**c.** to yield the substituted 2-(2-(2,4-dichlorophenyl)–1*H*-benzo[*d*]imidazol–1-yl)–*N*–(4–((*E*)–3–phenylacryloyl)phenyl)acetamide derivatives **90a**–**c**. The final compounds were screened for antimicrobial activity against methicillin-resistant *Staphylococcus aureus*, *Escherichia coli*, *Klebsiella pneumoniae*, *Pseudomonas aeruginosa*, *Acinetobacter baumannii*, *Candida albicans*, and *Cryptococcus neoformans* (Table 6) [52]. The compounds showed moderate activity against MRSA and *K*. *pneumoniae*.

**Scheme 17:**
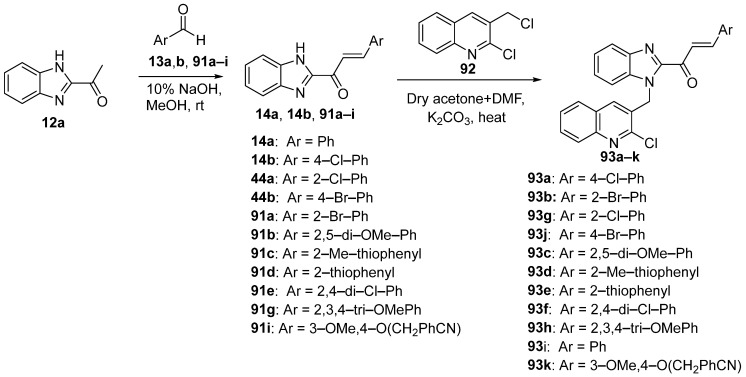
Synthesis of substituted (*E*)–1–(1–((2–chloroquinolin-3–yl)methyl)–1*H*–benzo[*d*]imidazol–2–yl)–3–phenylprop–2–en–1–one derivatives.

Karmur et al. reported the synthetic approach of the (*E*)–1–(1–((2–chloroquinolin–3-yl)methyl)–1*H*–benzo[*d*]imidazol–2–yl)–3–phenylprop–2–en–1–one derivatives (Scheme 17) [53], which was initiated from the union of 1–(1*H*–benzo[*d*]imidazol–2–yl)ethan–1–one **12a** with benzaldehyde **13a** to offer an intermediate (*E*)–1–(1*H*–benzo[*d*]imidazol–2–yl)–3–phenylprop–2–en–1–one **14a**–**b** and **91a**–**i**. Compounds **14a**, **14b**, and **91a**–**i** were further coupled with 2–chloro–3–(chloromethyl)quinoline (**92**) using Claisen–Schmidt condensation reaction to yield compounds **93a**–**k** in excellent yields (87–94%). The RAS activity was investigated through the antimicrobial screening, and the results revealed that several of these synthesized hybrids demonstrated significant antimicrobial potential, whereby the target compounds **93f**, **93g**, and **93h** were identified as the most active against *E*. *coli* and *P*. *aeruginosa*, demonstrating greater zones of inhibition compared to Tetracycline [53].

**Figure 6:**
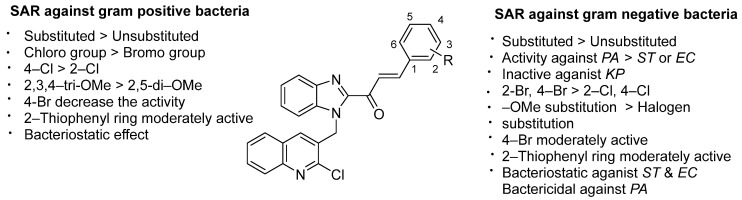
SAR of (*E*)–1–(1–((2–chloroquinolin–3–yl)methyl)–1*H*–benzo[*d*]imidazol–2–yl)–3–phenylprop-2–en–1–one derivatives.

Figure 6 shows a summary of SAR activity of the compounds. The benzimidazole–2–chloroquinoline hybrids with halogen and methoxy groups on phenyl ring showed greater potency for antimicrobial activity against Gram–positive bacteria. The results on the Gram-negative bacteria showed that compounds with substituents on the phenyl ring were more potent than the unsubstitued compounds. The methoxy group was essential for the activity, as the compounds with the methoxy functionalities were more active than the halogenated compounds [53].

### 3.3. Benzimidazole–Chalconewith Antiparasitic Activity

#### 3.3.1. Anti-Leishmanial C-2-Substituted Derivatives

**Scheme 18:**
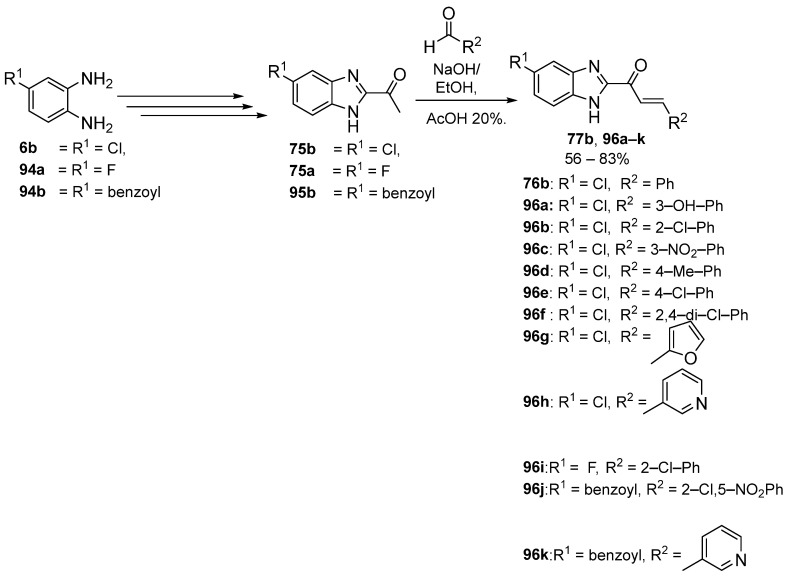
Synthesis of benzimidazole–chalcones.

The burden posed by Leishmaniasis, due to the limitations of current therapeutic options, has caused the need for preparing new and effective anti–leishmanial agents. The benzimidazole–chalcones, mostly with C–2 substituents, have shown activities against Leishmania parasites. N’Guessan et al. (2021) have reported a series of chloro–substituted benzimidazolyl–chalcone compounds **75b** and **96a**–**k**, which were tested against Leishmania parasite (Scheme 18) [54,55]. Here the chloro-substituted benzimidazolyl–chalcone compounds **75b** and **95a**–**j** were synthesized in three-steps. First the differently substituted *o*–phenylenediamines **6b** and **94** were condensed with 2–hyroxypropanoic acid under Phillip’s conditions to form the intermediates 2-hydroxyethylbenzimidazoles, which were subsequently oxidized with K_2_Cr_2_O_7_/H_2_SO_4_ to offer the substituted benzo[*d*]imidazol–2–ylethanones **75b** and **95a**. The synthesized compounds **75b** and **95a** were subjected to Claisen–Schmidt reaction conditions by condensing them with various aldehydes in ethanol using KOH as a base. After quenching the reaction with 20% acetic acid the benzimidazole–chalconederivatives **76b and 96a**–**j** were obtained in good yields (56–83%) [54,55].

The synthesized compounds were subjected to computational studies that include molecular docking, MMGBSA, and molecular dynamics, QSAR and ADME studies as anti–leishmanial targets, as well as in vitro anti–leishmanial activity. The results are shown in Table 7. Compounds **76b**, **96a**, and **96b** showed better anti-*Leishmania donovani* activities, with IC_50_ values ranging from 0.47 to 0.53 µM. The nitro derivative **96c** exhibited low activity with IC_50_ value of 74.45 ± 7.44 µM (Table 7) [54,55]. The results showed that the halogen substituent on the benzimidazole core was significant for the activity. The compounds with benzoyl substituent showed poor activity [54,55].

#### 3.3.2. Anti-Malarial Activity of C-2-Substituted Derivatives

**Scheme 19:**
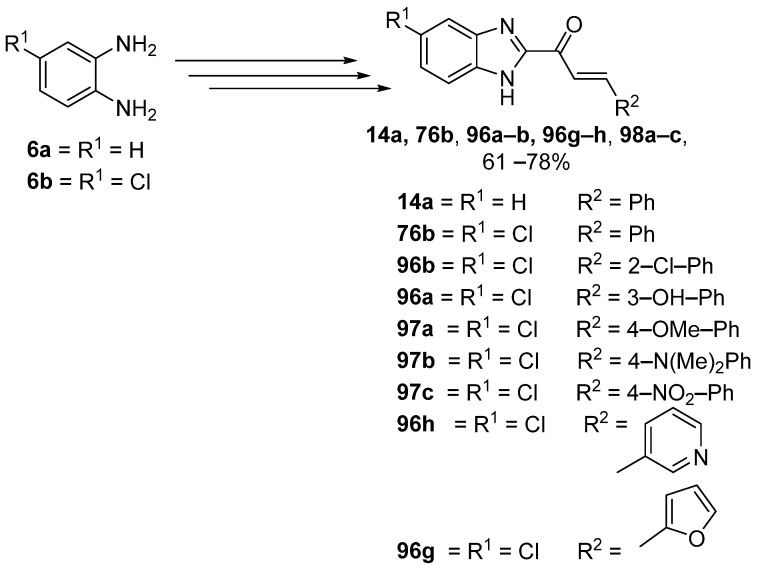
Synthesis of substituted benzimidazole–chalcones.

The spread of malaria, specifically in the Southern African countries has remained the significant challenge, demanding the urgent discovery of novel antimalarial compounds. In another study, N’Guessan et al. (2021) reported the synthesis of 5–chlorobenzimidazole–chalconederivatives, as the inhibitors of *Plasmodium falciparum* [54,55]. The synthesized compounds were obtained following the reported methodology (Scheme 19), whereby the synthesis was initiated from substituted *o*–phenylenediamines **6a** and **6b** to obtain targeted compounds **14a**, **76b**, **96a**–**b**, **96g**–**h**, **97a**–**c** [54,55].

The compounds were screened against chloroquine-sensitive (CQ–S) and chloroquine–resistant (CQ–R) strains of *P. falciparum* (Table 8). They expressed anti–plasmodial activities against chloroquine–resistant strain and showed a higher efficacy with the IC_50_ values ranging between 0.32 and 44.38 µM. The methoxylated derivative **97a** displayed the best antimalarial activity against CQ-S *P. falciparum* isolate with an IC_50_ of 0.32 ± 0.03 µM, and 1.96 ± 0.20 µM for the CQ-R *P*. *falciparum* isolate. On the other hand, the unsubstituted 5–chlorobenzimidazole derivative **76b** showed activity with the IC_50_ value of 0.78 ± 0.08 µM only for CQ–R *P. falciparum* [55].

### 3.4. Anti–SARS–CoV–2 Activity of C–2-Substituted Derivatives

**Figure 7:**
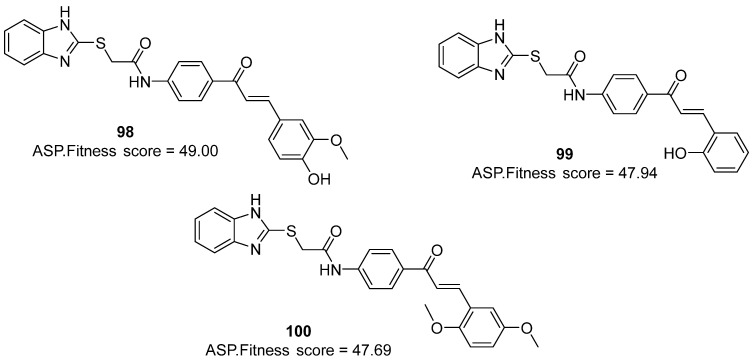
Some structural representative derivatives of 2-mercaptobenzimidazole.

The global threat posed by COVID-19 as a result of the SARS–CoV–2, has emphasized the urgent need for continued research into new antiviral scaffolds. Research on benzimidazole–chalcones with C–2 substituents has led to the identification of compounds with anti–SARS–CoV–2 activity. Porto et al. have reported compounds containing 2–mercaptobenzimidazole (2–MBI) conjoined with chalcone derivatives **98**, **99**, and **100** (Figure 7). The synthesized compounds were tested for inhibition of the spike protein of SARS–CoV–2 by screening them through ADMET predictions and molecular docking. The 2–MBI derivatives rose as potential antiviral alternatives in treating COVID–19. The compounds displayed a low oral acute toxicity class (LD_50_), engaging them as part of the Globally Harmonized System (GHS) classification for acute oral toxicity [56].

## 4. Chalcone–Benzimidazole Hybrids with Anti-Diabetic Activity

**Scheme 20:**
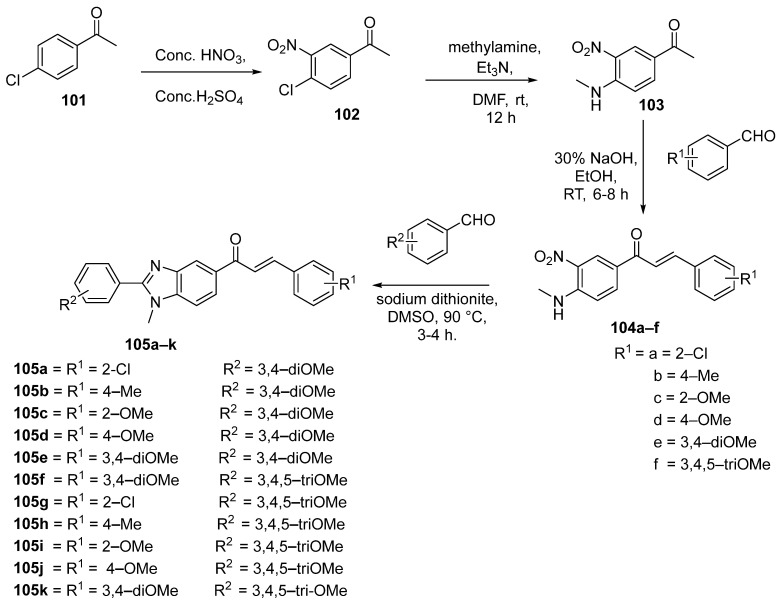
Synthesis of benzimidazole–chalconederivatives.

Benzimidazole–chalconehybrids serve as representative promising compounds, particularly for type 2 diabetes mellitus. Rai et al. described the synthesis of 5–substituted benzimidazole–chalcone scaffolds (Scheme 20), which were prepared by an efficient ‘one-pot’ nitro reductive cyclization [57]. The synthesis was initiated by the nitration of 4-chloroacetophenone (**101**) under acidic conditions offering 4–chloro–3–nitroacetophenone (**102**), which was further reacted with methylamine in the presence of triethylamine producing 1–(4–(methylamino)–3–nitrophenyl) ethan-1-one (**103**). The chalcones **104a**–**f** were obtained from the reaction of compound **104** with differently substituted benzaldehydes. The final chalcone–benzimidazoles **105a**–**k** were prepared by an efficient ‘one–pot’ nitro reductive cyclization of **104a**–**f** with either di or trimethoxylated aromatic aldehydes. The synthesized compounds were evaluated for α–glucosidase and α–amylase inhibition, and compound **105i** emerged as a potent antidiabetic agent with IC_50_ = 22.45 ± 0.36 µg/mL and 20.47 ± 0.60 µg/mL against α–glucosidase and α–amylase enzymes, respectively. Furthermore, it was found to be a safer candidate, which did not exert toxicity on the normal Human embryonic Kidney 293 (HEK293) cell line in comparison to standard Mitomycin [57].

## 5. Chalcone-Benzimidazoles with Monoamine Oxidase Inhibitory Activity

**Scheme 21:**
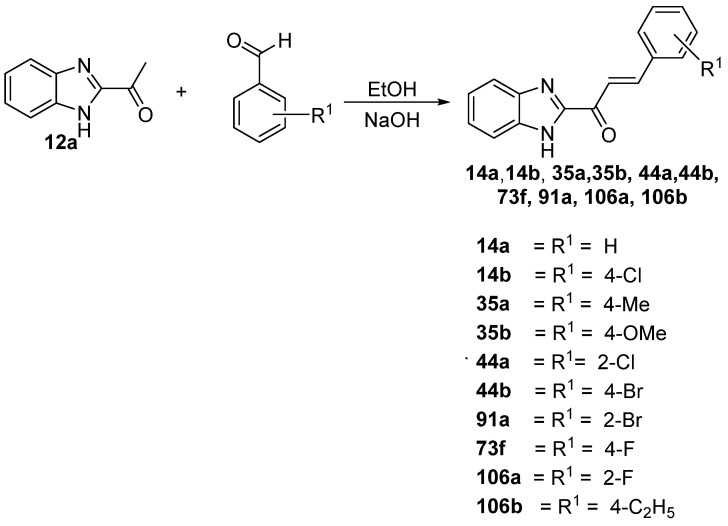
Synthesis of benzimidazole–chalconemonoamine oxidase inhibitors.

Research on benzimidazole–chalcone hybrids with monoamine oxidase (MAO) inhibitory activity is valuable since these molecules have demonstrated a promising opportunity for applications for both neurological and psychiatric disorders. Hence, these compounds could lead to the development of some effective MAO inhibitors with limited side–effects. The synthesis of benzimidazole–chalconederivatives **14a**, **14b**, **35a**,**35b**, **44a**, **44b**, **73f**, **91a**, **106a**–**b** was reported by Krishna et al. (2023) [58]. The compounds were prepared by reacting to the 1–(1*H*–benzo[*d*]imidazol–2–yl)ethanone (**12**) with various substituted benzaldehydes to achieve the targeted compound **14a**, **14b**, **35a**,**35b**, **44a**, **44b**, **73f**, **91a**, **106a**–**b** (Scheme 21). The synthesized compounds were screened for inhibitory activity against Monoamine oxidase B (MAO–B) and MAO–A. The results revealed that the compounds were potent against MAO–B than MAO–A, as shown in Table 9. The compound **44a** has shown good IC_50_ values of 0.80 ± 0.0094 µM for inhibition of MAO–B while for MAO–A a higher IC_50_ value of 35.29 ± 4.55 µM was recorded. The compound **106b** offered better results of 1.63 ± 0.10 and 2.04 ± 0.13 IC_50_ (µM) for MAO–A and MAO–B, respectively [58]. Most halogenated compounds showed better inhibitory activity on MAO–A and MAO–B than non-halogenated analogues [58].

## 6. Conclusions

The benzimidazole–chalcones contribute as bioactive synthetic compounds. Several groups have prepared benzimidazole–chalconederivatives that have displayed widespread of pharmacological properties. This review provided the advancements regarding the synthetic approaches and pharmacological properties of benzimidazole–chalconehybrids. The studies signify that benzimidazole and the chalcone moieties play a significant role in the development of biologically active compounds. Therefore, the compounds bearing benzimidazole and chalcone chromophores are worth to be synthesized based on the evidence from their in vitro and in silico studies. Future research should focus on in vivo studies of the benzimidazole–chalcones. Furthermore, the chalcone chromophore can further be derivatised into heterocyclic structures to expand the substrate scope. Target-based studies as well as mechanistic studies are also encouraged.

## Figures and Tables

**Table 1:** Cytotoxic activity of benzimidazole–chalcone against AW8507 and AW13516.

Compd. Code	R^1^	IC_50_ (μM) AW8507	IC_50_ (μM) AW13516
**14a**	Ph	–	–
**14b**	Ph–4–Cl	–	36.01
**15c**	Ph-3, 4–OMe	–	–
**14d**	Ph-4-NO_2_	7.89	7.12
**14e**	Ph-3-OMe	–	–
**14f**	4-Naphthyl	40.13	23.24
**14g**	2-Cl-Quinolinyl	–	–

R^1^ represent different substituents on the compounds.

**Table 2:** Antiproliferative activities of compounds **28a**–**o**.

Compd. Code	Cell Viability %	Antiproliferative Activity IC_50_ ± SEM (µM) ^a^			
		A–549	MCF–7	Panc–1	HT–29
**27a**	85	13.20 ± 1.60	13.90 ± 1.30	13.80 ± 1.40	13.90 ± 1.20
**27b**	84	6.50 ± 0.30	6.60 ± 0.60	7.45 ± 1.20	7.80 ± 0.40
**27c**	87	4.55 ± 0.60	3.62 ± 0.20	4.60 ± 0.90	4.80 ± 0.40
**27d**	91	10.70 ± 1.60	10.20 ± 1.60	10.60 ± 1.30	10.50 ± 1.20
**27e**	89	16.50 ± 1.20	17.10 ± 1.40	17.30 ± 1.20	16.90 ± 1.30
**27f**	89	2.97 ± 0.70	2.40 ± 1.00	3.20 ± 0.50	3.10 ± 0.90
**27g**	89	1.56 ± 0.20	2.10 ± 0.10	1.89 ± 0.20	1.92 ± 0.60
**27h**	96	1.30 ± 0.50	0.80 ± 0.08	1.20 ± 0.20	1.13 ± 0.20
**27i**	92	1.71 ± 0.60	1.92 ± 0.30	1.91 ± 0.50	2.21 ± 0.80
**27j**	89	1.92 ± 0.48	2.11 ± 0.20	2.12 ± 0.40	2.33 ± 0.80
**27k**	89	27.10 ± 3.10	26.70 ± 3.10	28.30 ± 3.20	27.90 ± 3.20
**27l**	89	21.80 ± 2.60	21.60 ± 2.80	22.10 ± 2.50	21.15 ± 2.60
**27m**	90	22.90 ± 2.80	23.20 ± 2.80	24.05 ± 3.20	23.75 ± 2.60
**27n**	87	26.90 ± 2.50	26.40 ± 2.10	28.10 ± 2.20	27.40 ± 2.20
**27o**	89	22.80 ± 2.50	22.20 ± 2.50	23.45 ± 3.10	24.15 ± 2.50

^a^ SEM = Standard Error of the Mean.

**Table 3:** Antiproliferative activities of compounds **28a**–**k**.

Compd. Code	Cell Viability %	Antiproliferative Activity IC_50_ ± SEM (µM) ^a^			
		A–549	MCF–7	Panc–1	HT–29
**28a**	90	14.80 ± 1.50	14.70 ± 1.40	15.50 ± 1.20	16.10 ± 1.10
**28b**	86	4.90 ± 0.50	4.75 ± 0.40	5.50 ± 0.20	6.50 ± 1.10
**28c**	90	12.70 ± 1.60	11.90 ± 1.40	11.80 ± 1.30	12.10 ± 1.40
**28d**	84	21.40 ± 2.70	20.60 ± 1.80	20.70 ± 3.50	19.85 ± 3.60
**28e**	87	3.00 ± 0.50	3.50 ± 0.20	3.40 ± 0.20	3.60 ± 0.40
**28f**	90	1.67 ± 0.60	1.91 ± 0.30	1.82 ± 0.50	2.17 ± 0.80
**28g**	86	1.67 ± 0.60	0.96 ± 0.08	1.52 ± 0.20	1.63 ± 0.20
**28h**	89	1.77 ± 0.68	2.01 ± 0.40	1.98 ± 0.50	2.27 ± 0.80
**28i**	87	2.41 ± 0.40	2.72 ± 0.30	2.81 ± 0.50	2.71 ± 0.80
**28j**	88	29.30 ± 2.50	28.90 ± 2.80	28.50 ± 2.50	28.90 ± 2.50
**28k**	85	28.90 ± 2.50	28.40 ± 2.10	29.10 ± 2.50	28.40 ± 2.50
Doxorubicin	-	1.21 ± 0.80	0.90 ± 0.62	1.41 ± 0.58	1.01 ± 0.82

^a^ SEM = Standard Error of the Mean.

**Table 4:** Selected benzimidazole hybrid compounds and their cytotoxic activities in vitro ^a^ (IC_50_, μM ^b^).

Compd. Code	Benzimidazole Ring (R^1^)	R^2^	HL-60	SMMC-7721	A-549	MCF–7	SW480
**65a**	5,6–methyl–benzimidazole	–	1.95	3.90	5.87	4.84	5.10
**65b**	2–methyl–benzimidazole	–	1.08	2.50	2.28	3.56	2.90
**65c**	benzimidazole	–	1.69	2.16	2.19	3.48	2.95
**67a**	5,6–dimethyl-benzimidazole	4-OMe–phenacyl	1.18	7.51	>20	2.90	6.85
**67b**	5,6–dimethyl-–benzimidazole	4-Br–benzyl	2.23	7.23	12.68	5.49	8.10
**67c**	5,6–dimethyl–benzimidazole	2-Br–benzyl	1.93	6.57	13.32	4.38	7.69
**67d**	5,6–dimethyl–benzimidazole	naphthylmethyl	0.83	6.35	7.97	1.57	2.92
**67e**	5,6–dimethyl–benzimidazole	benzyl	4.19	7.16	12.26	5.44	7.88
**67f**	5,6–dimethyl–benzimidazole	4–Me-benzyl	0.59	5.92	8.15	2.83	7.31
**68a**	2–methyl–benzimidazole	4–OMe-phenacyl	2.00	5.64	11.18	2.49	4.68
**68b**	2–methyl–benzimidazole	phenacyl	4.73	5.28	11.38	2.42	5.01
**68c**	2–methyl–benzimidazole	4–Br–phenacyl	1.21	5.89	11.75	3.07	5.51
DPP			2.11	11.27	6.94	17.43	17.05

^a^ Data represents the mean values of three independent determinations. ^b^ Cytotoxicity as IC_50_ for each cell line. Cisplatin (DDP). R2 represent different substituents on the compounds.

**Table 5:** In vitro antifungal activities of **10a**, **76a**–**d** and reference substances with respect to Candida albicans.

Compd. Code	R	Anticandidosic Screening (MIQ)	*Candida albicans* MIC (µg/mL)
**14a**	H	+	5
**76a**	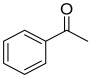	−	Nd
**76b**	Cl	+	1.25
**76c**	F	+	5
**76d**	NO_2_	−	Nd

(+): Active (QMI = 10 µg), (−): Inactive (QMI > 10 µg).

**Table 6:** Antimicrobial activity (growth inhibition % at 32 μg mL^−1^ concentration) of the examined compounds.

Compd. Code	Gram-Positive Bacteria	Gram-Negative Bacteria			Fungi		
	MRSA ^a^	Ec ^b^	Kp ^c^	Pa ^d^	Ab ^e^	Ca ^f^	Cn ^g^
	GI%						
**90a**	31.19	2.46	32.67	10.17	16.21	−8.96	−12.29
**90b**	18.97	−4.20	22.36	7.57	14.73	1.42	−1.06
**90c**	26.81	−1.30	31.76	7.57	9.46	−14.22	−38.14

^a^ Methicillin-resistant *Staphylococcus aureus*. ^b^ *Escherichia coli*. ^c^ *Klebsiella pneumoniae*. ^d^ *Pseudomonas aeruginosa*. ^e^ *Acinetobacter baumannii*. ^f^ *Candida albicans*. ^g^ *Cryptococcus neoformans*.

**Table 7:** Benzimidazolyl–chalcones derivatives and their anti–*L. donovani* activities.

Compd. Code	IC_50_ (mg/mL)	IC_50_ µM
**76b**	0.15	0.53 ± 0.05
**96a**	0.15	0.50 ± 0.05
**96b**	0.15	0.47 ± 0.04
**96c**	24.4	74.45 ± 7.44
**96d**	0.31	1.04 ± 0.10
**96e**	7.80	24.59 ± 2.46
**96f**	0.63	1.79 ± 0.18
**96g**	0.31	1.14 ± 0.11
**96h**	3.90	13.79 ± 1.38
**96i**	0.40	1.33 ± 0.13
**96j**	45.70	100.80 ± 10.08
**96k**	18.60	43.07 ± 4.30

**Table 8:** Median IC_50_ of all tested compounds by type of isolates.

Compd. Code	Chloroquine Sensitive *P. falciparum* Isolate IC_50_ (µM) ±SD	Chloroquine Resistant *P. falciparum* Isolate IC_50_ (µM) ± SD
Chalcone	38.56 ± 3.86	1.44 ± 0.14
**14a**	44.38	6.81
**76b**	10.65 ± 1.07	0.78 ± 0.08
**96b**	32.10 ± 3.21	31.28 ± 3.13
**96a**	6.23 ± 0.62	2.34 ± 0.23
**97a**	0.32 ± 0.03	1.96 ± 0.20
**97b**	9.40 ± 0.94	1.82 ± 0.18
**97c**	29.39 ± 2.94	10.61 ± 0.11
**96h**	29.61 ± 2.96	22.42 ± 2.24
**96g**	–	9.34 ± 0.93
Chloroquine	0.076 ± 0.008	0.13 ± 0.013

**Table 9:** The inhibitions of MAO–A and B IC_50_ (µM).

Compd Code	R^1^	Inhibitions of MAO–A IC_50_ (µM)	Inhibitions of MAO–B IC_50_ (µM)
**14a**	H	>40	>40
**14b**	4–Cl	5.59 ± 1.12	4.83 ± 0.76
**35a**	4–Me	>40	10.02 ± 0.30
**35b**	4–OMe	>40	11.09 ± 0.07
**44a**	2–Cl	35.29 ± 4.55	0.80 ± 0.0094
**44b**	4–Br	8.39 ± 0.70	6.87 ± 1.79
**73f**	4–F	>40	10.94 ± 0.89
**91a**	2–Br	27.17 ± 0.14	1.11 ± 0.17
**106a**	2–F	1.63 ± 0.10	2.04 ± 0.13
**106b**	4–C_2_H_5_	>40	8.32 ± 1.53

R^1^ represent different substituents on the compounds.

## Data Availability

Not Applicable.

## References

[1] Rammohan A., Reddy J.S., Sravya G., Rao C.N., Zyryanov G.V. (2020). Chalcone synthesis, properties and medicinal applications: A review. Environ. Chem. Lett..

[2] Elkanzi N.A., Hrichi H., Alolayan R.A., Derafa W., Zahou F.M., Bakr R.B. (2022). Synthesis of chalcones derivatives and their biological activities: A review. ACS Omega.

[3] Jasim H.A., Nahar L., Jasim M.A., Moore S.A., Ritchie K.J., Sarker S.D. (2021). Chalcones: Synthetic chemistry follows where nature leads. Biomolecules.

[4] Michalkova R., Mirossay L., Gazdova M., Kello M., Mojzis J. (2021). Molecular mechanisms of antiproliferative effects of natural chalcones. Cancers.

[5] Zhou Q., Tang X., Chen S., Zhan W., Hu D., Zhou R., Sun N., Wu Y., Xue W. (2022). Design, synthesis, and antifungal activity of novel chalcone derivatives containing a piperazine fragment. J. Agric. Food Chem..

[6] Pérez-González A., Castañeda-Arriaga R., Guzmán-López E.G., Hernández-Ayala L.F., Galano A. (2022). Chalcone derivatives with a high potential as multifunctional antioxidant neuroprotectors. ACS Omega.

[7] de Oliveira A.S., Cenci A.R., Gonçalves L., Thedy M.E.C., Justino A., Braga A.L., Meier L. (2024). Chalcone derivatives as antibacterial agents: An updated overview. Curr. Med. Chem..

[8] Zhan W., Mao P., Yuan C., Zhang Y., Zhang T., Liu Y., Tian J., Xue W. (2023). Design, synthesis and antiviral activities of chalcone derivatives containing pyrimidine. J. Saudi Chem. Soc..

[9] Osman M.S., Awad T.A., Shantier S.W., Garelnabi E.A., Osman W., Mothana R.A., Nasr F.A., Elhag R.I. (2022). Identification of some chalcone analogues as potential antileishmanial agents: An integrated in vitro and in silico evaluation. Arab. J. Chem..

[10] Okolo E.N., Ugwu D.I., Ezema B.E., Ndefo J.C., Eze F.U., Ezema C.G., Ezugwu J.A., Ujam O.T. (2021). New chalcone derivatives as potential antimicrobial and antioxidant agent. Sci. Rep..

[11] Guazelli C.F., Fattori V., Ferraz C.R., Borghi S.M., Casagrande R., Baracat M.M., Verri W.A. (2021). Antioxidant and anti-inflammatory effects of hesperidin methyl chalcone in experimental ulcerative colitis. Chem. Biol. Interact..

[12] Constantinescu T., Lungu C.N. (2021). Anticancer activity of natural and synthetic chalcones. Int. J. Mol. Sci..

[13] Sinha S., Medhi B., Radotra B., Batovska D.I., Markova N., Bhalla A., Sehgal R. (2022). Antimalarial and immunomodulatory potential of chalcone derivatives in experimental model of malaria. BMC Complement. Med. Ther..

[14] Sekar P., Kumar S., Raju S.K. (2023). An Updated Review on Recent Advancements in the Diverse Biological Applications of Medicinally Privileged Scaffold: Chalcone and its derivatives. Int. J. Med. Sci. Pharma Res..

[15] Amin M.M., Abuo-Rahma G.E.-D.A., Shaykoon M.S.A., Marzouk A.A., Abourehab M.A., Saraya R.E., Badr M., Sayed A.M., Beshr E.A. (2023). Design, synthesis, cytotoxic activities, and molecular docking of chalcone hybrids bearing 8-hydroxyquinoline moiety with dual tubulin/EGFR kinase inhibition. Bioorg. Chem..

[16] Upendranath K., Venkatesh T., Nayaka Y.A., Shashank M., Nagaraju G. (2022). Optoelectronic, DFT and current-voltage performance of new Schiff base 6-nitro-benzimidazole derivatives. Inorg. Chem. Commun..

[17] Rao A.M., Kumar T.A., Devi B.R. (2016). Synthesis of certain benzimidazoloquinolone carboxylic derivatives as potential antitubercular agents. Rasayan J. Chem..

[18] Hobrecker F. (1872). Reduction-products of nitracetamide compounds. Ber. Dtsch. Chem. Ges..

[19] Ladenburg A. (1875). Derivate von diaminen. Ber. Dtsch. Chem. Ges..

[20] Tahlan S., Kumar S., Narasimhan B. (2019). Pharmacological significance of heterocyclic 1H-benzimidazole scaffolds: A review. BMC Chem..

[21] Anand K., Wakode S. (2017). Development of drugs based on benzimidazole heterocycle: Recent advancement and insights. Int. J. Chem. Stud..

[22] Sharma P., Reddy T.S., Thummuri D., Ram K., Praveen N., Naidu V.G.M., Bhargava S.K., Shankaraiah N. (2016). Synthesis and biological evaluation of new benzimidazole- thiazolidinedione hybrids as potential cytotoxic and apoptosis inducing agents. Eur. J. Med. Chem..

[23] Venkateswarlu Y., Kumar S.R., Leelavathi P. (2013). Facile and efficient one-pot synthesis of benzimidazoles using lanthanum chloride. Bioorg. Med. Chem. Lett..

[24] Alzahrani H.A., Alam M.M., Elhenawy A.A., Nazreen S. (2022). Synthesis, Antimicrobial, Antiproliferative, and Docking Studies of 1, 3, 4-Oxadiazole Derivatives Containing Benzimidazole Scaffold. Biointerface Res. Appl. Chem..

[25] Romero J.A., Acosta M.E., Gamboa N.D., Mijares M.R., De Sanctis J.B., Llovera L.J., Charris J.E. (2019). Synthesis, antimalarial, antiproliferative, and apoptotic activities of benzimidazole-5-carboxamide derivatives. Med. Chem. Res..

[26] Leshabane M., Dziwornu G.A., Coertzen D., Reader J., Moyo P., van der Watt M., Chisanga K., Nsanzubuhoro C., Ferger R., Erlank E. (2021). Benzimidazole derivatives are potent against multiple life cycle stages of Plasmodium falciparum malaria parasites. ACS Infect..

[27] Satija G., Sharma B., Madan A., Iqubal A., Shaquiquzzaman M., Akhter M., Parvez S., Khan M.A., Alam M.M. (2022). Benzimidazole based derivatives as anticancer agents: Structure activity relationship analysis for various targets. J. Heterocycl. Chem..

[28] Mezgebe K., Melaku Y., Mulugeta E. (2023). Synthesis and pharmacological activities of chalcone and its derivatives bearing N-heterocyclic scaffolds: A review. ACS Omega.

[29] Janaki P., Sekar K., Thirunarayanan G. (2016). Synthesis, spectral correlation and insect antifeedant activities of some 2-benzimidazole chalcones. J. Saudi Chem. Soc..

[30] Gao F., Huang G., Xiao J. (2020). Chalcone hybrids as potential anticancer agents: Current development, mechanism of action, and structure-activity relationship. Med. Res. Rev..

[31] Mahama O., Aboudramane K., Soleymane K., Sylvain C., Sekou D., Drissa S. (2020). Anticancer Activities and QSAR Study of Novel Agents with a Chemical Profile of Benzimidazolyl-Retrochalcone. Open J. Med. Chem..

[32] Pool W., Harwood H., Ralston A. (1937). 2-Alkylbenzimidazoles as derivatives for the identification of aliphatic acids. J. Am. Chem. Soc..

[33] Koné A., Souleymane C., Kalo M., Tchambaga Etienne C., Collet S., Sissouma D. (2025). Synthesis of novel benzimidazole-based retrochalcones and their anticancer activity against breast and colon cancer. Synth. Commun..

[34] Kone A., Ouattara M., Zon D., Chany A., Collet S., Sissouma D., Adjou A. (2018). Synthesis and Cytotoxic Activities of 3-Benzimidazolyl-Chalcones Derivatives. World J. Pharm. Res..

[35] Uthale A., Anantram A., Sulkshane P., Degani M., Teni T. (2023). Identification of bicyclic compounds that act as dual inhibitors of bcl-2 and mcl-1. Mol. Divers..

[36] Hagar F.F., Abbas S.H., Abdelhamid D., Gomaa H.A., Youssif B.G., Abdel-Aziz M. (2023). New 1, 3, 4-oxadiazole-chalcone/benzimidazole hybrids as potent antiproliferative agents. Arch. Pharm..

[37] Pragathi Y.J., Veronica D., Anitha K., Rao M.V.B., Raju R.R. (2020). Synthesis and biological evaluation of chalcone derivatives of 1, 2, 4-thiadiazol-benzo [d] imidazol-2-yl) quinolin-2 (1H)-one as anticancer agents. Chem. Data Collect..

[38] Farag B., Zaki M.E., Elsayed D.A., Gomha S.M. (2025). Benzimidazole chemistry in oncology: Recent developments in synthesis, activity, and SAR analysis. RSC Adv..

[39] Ibrahim A.A., Said E.G., AboulMagd A.M., Amin N.H., Abdel-Rahman H.M. (2025). Synthesis and SARs of benzimidazoles: Insights into antimicrobial innovation (2018–2024). RSC Adv..

[40] Hsieh C.-Y., Ko P.-W., Chang Y.-J., Kapoor M., Liang Y.-C., Chu H.-L., Lin H.-H., Horng J.-C., Hsu M.-H. (2019). Design and synthesis of benzimidazole-chalcone derivatives as potential anticancer agents. Molecules.

[41] Djemoui A., Naouri A., Ouahrani M.R., Djemoui D., Lahcene S., Lahrech M.B., Boukenna L., Albuquerque H.M., Saher L., Rocha D.H. (2020). A step-by-step synthesis of triazole-benzimidazole-chalcone hybrids: Anticancer activity in human cells^+^. J. Mol. Struct..

[42] Nawareg N.A., Mostafa A.S., El-Messery S.M., Nasr M.N. (2022). New benzimidazole based hybrids: Synthesis, molecular modeling study and anticancer evaluation as TopoII inhibitors. Bioorg. Chem..

[43] Zhou W., Zhang W., Peng Y., Jiang Z.-H., Zhang L., Du Z. (2020). Design, synthesis and anti-tumor activity of novel benzimidazole-chalcone hybrids as non-intercalative topoisomerase II catalytic inhibitors. Molecules.

[44] Ameji J.P., Ebune A.O., Aderemi I.W., Moyosore A., Idah G. (2023). Theoretical investigation and design of novel anti-proliferative agents against hepatocellular carcinoma from benzimidazole-chalcone derivatives. Adv. J. Chem. A.

[45] Yang J.-L., Ma Y.-H., Li Y.-H., Zhang Y.-P., Tian H.-C., Huang Y.-C., Li Y., Chen W., Yang L.-J. (2019). Design, synthesis, and anticancer activity of novel trimethoxyphenyl-derived chalcone-benzimidazolium salts. ACS Omega.

[46] Chhajed S.S., Sonawane S.S., Upasani C.D., Kshirsagar S.J., Gupta P.P. (2016). Design, synthesis and molecular modeling studies of few chalcone analogues of benzimidazole for epidermal growth factor receptor inhibitor in search of useful anticancer agent. Comput. Biol. Chem..

[47] Babu I.S., Selvakumar S. (2013). An antibacterial, antifungal and anthelmintic evaluations of some synthesized chalcone derived benzimidazoles. BBRA.

[48] Shahin M., AbdAlhafiz A., Abouzid K., Mehanna W. (2022). Synthesis, biological evaluation and in-silico ADME study of novel hybridized benzimidazole-chalcone compounds with potential antimicrobial activity. Arch. Pharm. Sci. A.

[49] Songuigama C., Jean-Paul N., Mamidou K.W., Drissa S., Mahama O. (2018). Anti-Candidosi Activities of New Chalcones Vectorised by Benzimidazole against a Strain of Candida albicans Pharmacoresistance to Azoles. J. Pharm. Biol. Sci..

[50] Parikh K., Joshi D. (2013). Antibacterial and antifungal screening of newly synthesized benzimidazole-clubbed chalcone derivatives. Med. Chem. Res..

[51] Meshram G.A., Vala V.A. (2015). Synthesis, characterization, and antimicrobial activity of benzimidazole-derived chalcones containing 1, 3, 4-oxadiazole moiety. Chem. Heterocycl. Compd..

[52] Ibrahim A.A., Said E.G., AboulMagd A.M., Amin N.H., Abdel-Rahman H.M. (2025). Novel benzimidazole hybrids: Design, synthesis, mechanistic studies, antifungal potential and molecular dynamics. RSC Med. Chem..

[53] Karmur M.B., Rakholiya K., Kaneria M., Karmur S.B., Bapodra A., Patel R., Maliwal D., Pissurlenkar R.R., Kapuriya N.P., Bhalodia J. (2025). Synthesis and Characterization of Some New Hybrids of 2-Chloroquinoline-Benzimidazole Chalcones as Potential Antibacterial Agents. ChemistrySelect.

[54] N’guessan D.U.J.-P., Alzain A.A., Adouko E.M., Sékou D., Coulibaly S., Ouattara M. (2021). Molecular modeling studies of benzimidazolyl-chalcones as antileishmanial agents using qsar, docking, ADME and molecular dynamics studies. J. Appl. Pharm. Sci. Res..

[55] N’Guessan D., Kablan L., Kacou A., Bories C., Coulibaly S., Sissouma D., Loiseau P., Ouattara M. (2021). Synthesis and biological profiles of some benzimidazolyl-chalcones as anti-leishmanial and trypanocidal agents. Chem. Sci. Int. J..

[56] Porto R.S., de Lima Costa L.F., Porto V.A. (2022). Identification of Synthetic 2-Mercaptobenzimidazole Derivatives as Inhibitors of Spike Protein of SARS-CoV-2 by Virtual Screening. Lett. Appl. NanoBioSci..

[57] Rai P.V., Ramu R., Akhileshwari P., Prabhu S., Prabhune N.M., Deepthi P., Anjana P., Ganavi D., Vijesh A., Goh K.W. (2024). Novel Benzimidazole-endowed chalcones as α-glucosidase and α-amylase inhibitors: An insight into structural and computational studies. Molecules.

[58] Krishna A., Lee J., Kumar S., Sudevan S.T., Uniyal P., Pappachen L.K., Kim H., Mathew B. (2023). Inhibition of monoamine oxidases by benzimidazole chalcone derivatives. Appl. Biol. Chem..

